# Anti-carcinogenic Effects of the Flavonoid Luteolin 

**DOI:** 10.3390/molecules13102628

**Published:** 2008-10-22

**Authors:** Günter Seelinger, Irmgard Merfort, Ute Wölfle, Christoph M. Schempp

**Affiliations:** 1 Medical and Pharmaceutical Services, Berlin, Germany; E-mail: dr.g.seelinger@arcor.de; 2 Institute of Pharmaceutical Sciences, Department of Pharmaceutical Biology and Biotechnology, University of Freiburg, Germany; E-mail: irmgard.merfort@pharmazie.uni-freiburg.de; 3 Competence center *skin*tegral, Department of Dermatology, University Medical Center Freiburg, Germany; E-mail: ute.woelfle@uniklinik-freiburg.de

**Keywords:** Luteolin, Cancer, Pharmacology, Epidemiology

## Abstract

Luteolin is a flavonoid which is part of our daily nutrition in relatively low amounts (less than 1 mg/day). Nevertheless, some epidemiological studies suggest an inverse correlation between luteolin intake and the risk of some cancer types. Luteolin displays specific anti-inflammatory and anti-carcinogenic effects, which can only partly be explained by its anti-oxidant and free radical scavenging capacities. Luteolin can delay or block the development of cancer cells *in vitro* and *in vivo* by protection from carcinogenic stimuli, by inhibition of tumor cell proliferation, by induction of cell cycle arrest and by induction of apoptosis via intrinsic and extrinsic signaling pathways. When compared to other flavonoids, luteolin was usually among the most effective ones, inhibiting tumor cell proliferation with IC_50_ values between 3 and 50 µM *in vitro* and *in vivo* by 5 to 10 mg/kg i.p., intragastric application of 0.1–0.3 mg/kg/d, or as food additive in concentrations of 50 to 200 ppm. Luteolin has been shown to penetrate into human skin, making it also a candidate for the prevention and treatment of skin cancer.

## 1. Introduction

There is good evidence suggesting that flavonoids contribute to the cancer-protective effect of fruits and vegetable food [1]. Flavonoids as free radical scavengers protect organisms from carcinogenic reactive oxygen species (ROS) and other radicals. Flavonoids are known to display anti-inflammatory capacities, an important feature because inflammatory processes are involved in cancer development, especially when they are long-lasting or excessive and destroy epithelial barriers. However, the differential analysis of the various flavonoids’ specific roles and potentials is just beginning. 

While quercetin has been studied most intensively among the flavonoids during the last decades, recent research has provided a plethora of anti-oxidant, immunological, and anti-carcinogenic mechanisms which suggest luteolin might be a valuable compound in anti-cancer strategies. Luteolin is a flavone contained in many medical herbs and in vegetables (e.g. parsley, artichoke, celery, green pepper, and perilla leaf). Luteolin concentrations in food are generally low compared to some of the flavonols like quercetin or kaempferol, but high amounts are found e.g. in peanut hulls and in *Reseda luteola L.*, the Dyer's weld, and may be made available at low costs. 

As other flavonoids, luteolin is most often found in plant materials in the form of glycosides, which are eventually metabolized by intestinal bacteria, cleaved and glucuronated during uptake in the gut and metabolized in the organism. Thus, *in vitro* investigations with pure luteolin or its naturally occurring glycosides have to be interpreted with care.

The present paper gives an account of cancer-related pharmacological and epidemiological data for luteolin and compares it to other flavonoids. Literature search was initiated by the end of February 2008 using “Luteolin” as a key-word in the PubMed database, yielding 1,085 results, and in Medline, with 1,077 citations. Restriction to title search in Medline gave 163 results, and 192 were obtained from PubMed under restriction to the toxicology section. Both lists were checked for relevant literature. Additionally, “related articles” proposed by PubMed for the selected citations were systematically searched. 

## 2. Flavonoids: some functionally relevant aspects of molecular structure

**Figure 1:**
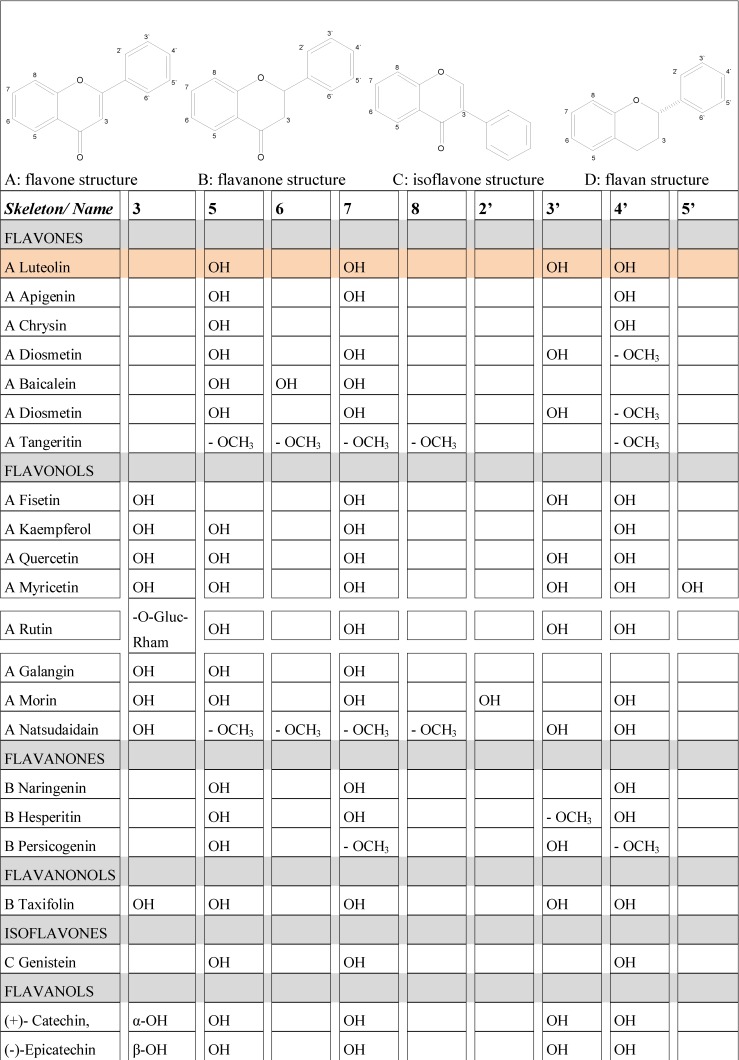
Substitution patterns of flavonoids which are mentioned in the text. Flavonoids are characterized by two aromatic rings connected by a C3 bridge and substitutions of hydroxyl groups or other moieties which influence their biological activity.

Flavonoids are characterized by a molecular frame of two phenyl rings linked by a three carbon chain, making them good electron donators or acceptors. Their anti-oxidant capacity depends on this framework, the number and pattern of substitutions (primarily with hydroxyl groups), their ability to chelate with metal ions, and on their specific environment. Anti-oxidant properties of a specific substance are complex, and relative efficacies of two substances can vary in different test assays [2]; [3]. A more comprehensive review of luteolin as an anti-oxidant, radical-scavenging and anti-inflammatory agent is published separately in a related paper [4]. Briefly, the *ortho*-dihydroxy structure in the B-ring and the 2,3-double bond in conjugation with the 4-oxo function of the C-ring provides a good, but not excellently high anti-oxidant capacity of luteolin. In cell-free tests, luteolin is usually inferior to quercetin which has an additional hydroxyl substitution in position 3 (see Figure 1 for terminology). However, it is more lipophilic and may perform better in test systems with biological molecules or membranes. Luteolin can form chelates with metal ions, but is again less active in this respect than quercetin [5]. However, it is not oxidized during the chelation process. This may explain why luteolin does not undergo redox cycling as do quercetin and other flavonols, a process increasing their potential to act as pro-oxidants with possible deleterious effects. Nevertheless, although structure activity relationship studies are a helpful tool to predict biological activities, experiments have to be carried out under physiological conditions for a final proof.

In plants, many flavonoids are found in the form of glycosides but they are cleaved to their aglycones in the intestinal mucosa, and the aglycones are degraded or glucuronated by UDP-glucuronosyl transferases before release into blood serum [6] Wittemer *et al.* [7] investigated pharmacokinetics of aqueous artichoke extracts containing luteolin-7-*O*-glucoside in humans. Neither luteolin nor its glucosides were found in urine or plasma, but only their phase II-conjugates.

Anti-oxidant properties are to a large extent attributable to hydroxylation in positions 3' and 4'; it is there, where flavonoids are preferentially glucuronidized during resorption in the gut [8]. This might reduce the flavonoids' anti-oxidant potential. However, several human tissues and cell lines, e.g., neutrophile granulocytes and CaCo-2 cells, are able to cleave luteolin glucuronides, and increase their activity when stimulated by pro-inflammatory substances [9]. 

*In vivo* skin penetration studies of the flavones apigenin, luteolin, and apigenin-7-*O*-β-glucoside with human volunteers showed that except of the glucoside they were not only adsorbed at skin surface, but penetrated into deeper skin layers. This is important for their topical use as antiphlogistic agents in dermatology [10].

## 3. Pharmacological Effects

Anti-cancer-strategies include protection of tissue from carcinogenic stimuli, suppression of pro-carcinogenic regulatory mechanisms and cell proliferation, modulation of intercell communication signals, destruction or removal of tumor cells, and induction of apoptosis. In solid tumors, a further strategy is to inhibit growth of blood vessels which supply and promote the tumor. Luteolin, like a number of other flavonoids and polyphenols [11] displays a variety of pharmacological effects *in vitro* and *in vivo* which may contribute to anti-carcinogenic activity provided that effective concentration levels are reached at the target sites under realistic conditions. Since bioavailability of flavonoids is a critical matter and needs more quantitative investigations, special attention is paid to effective concentrations in this review. Table 1 gives an account of the anti-carcinogenic effects and mechanisms reported for luteolin.

**Table 1:** Anti-cancer effects and mechanisms investigated with luteolin.

Target	Effect of luteolin	Publication source(s)
Tumor cell proliferation	Inhibits proliferation of various tumor cell lines *in vitro*	[12,13,14,15,16,17,18,19,20,21,22,23,25,27,28,30,31,32,34,35]
Cell cycle arrest	Induces cell cycle arrest in G2/M, S, or G0/1 phase	[19,22,23,24,25,26]
Angiogenesis	Inhibits bFGF and VEGF induced *in vitro* angiogenesis of BBE cells	[12]
Tumor growth *in vivo*	Inhibits growth of tumors from different organs when applied p.o. or i.p.	[21,33,34,35,36,37,38,40,50,68]
Anti-oxidant enzymes and compounds	Increases levels of SOD, CAT, GPx, Vit A, Vit C, ß-Carotene	[29,49,50,51]
NAT	Inhibits N-acetyltransferase in cancer cell lines	[54,56,57]
MMP	Inhibits secretion of MMP-2 and MMP-9 release	[20]
IGF / IFR	Inhibits tyrosin phosphorylation of IGFR	[36]
EGF / EGFR	Inhibits EGF or IGF-1 induced activation of EGFR	[20,36,66]
HGF / c-Met	Inhibits phosphorylation of hepatocyte growth factor receptor c-Met	[58]
AR	Downregulates androgen receptor	[35]
PI-3-Kinase / Akt	Inhibits Akt phosphorylation	[36,65,66,67]
PI-3-Kinase / Akt	Suppresses Akt expression	[21]
Mitochondria	Reduces mitochondrial membrane potential	[21,70]
Cytochrome C	Induces cytochrome C release into cytosol	[21,70,71]
Bcl-2 family	Increases Bax, Bad, Bax / Bcl2 ratio; decreases Bcl2, increases Bax / Bak translocation	[21,23,25,34,70]
AI-P	Induces expression of apoptose-inducing factor	[26]
Caspase 9	Activates caspase 9	[21,23,70,72,73]
Caspase 3 / 6 / 7	Activates caspase 3	[21,23,25,26,70,73]
Caspase 8 / 10	Activates Caspase 8 / 10 via death receptors	[68,72]
PARP	Cleaves poly (ADP-ribose) polymerase	[21,26,70]
DFF-45	Activates DNA fragmentation factor	[70]
Topoisomerase	Inhibits topoisomerase I and II	[60,61,64]
MAPK / ERK	Inhibits IGF induced activation of signaling; suppresses ERK expression	[21,36]
JNK / p38	Activates JNK	[68,71]
p21	Increases expression of p21 in p53 knockout cells	[36]
p21	low doses induce, high doses suppress p21 expression	[23,33]
p53	Stabilizes p53 via JNK activation; Accumulates p53	[68] [24]
Cyclin	Inhibits expression of cyclin D1 induced by IGFR or pSTAT3	[34,36]
survivin	Downregulates survivin	[23,34]
PCNA	Reduces expression of proliferating cell nuclear antigen	[21]
Fas/CD95	Increases Fas/CD95 expression	[34]
TRAIL	Sensitizes TRAIL-induced apoptosis	[68]
XIAP	Reduces X-linked inhibitor of apoptosis protein	[74]
FASN	Inhibits fatty acid synthesis in cancer cells	[75]

### 3.1 Inhibition of tumor cell proliferation

#### 3.1.1 *In vitro*

Fotsis *et al.* [12] reported that certain flavonoids are potent inhibitors of various human normal (fibroblasts HFK2, keratinocytes HaCaT) and cancer (breast cancer MCF-7, neuroblastoma-derived SHEP and WAC2) cell lines in the low micromolar range. Luteolin was effective in all cell lines and was among the most potent inhibitors (IC_50_ concentrations between 1.1 µM and 7.6 µM) together with hydroxyflavone, 3',4'-dihydroxyflavone, 2',3'-dihydroxyflavone, fisetin, and apigenin. These flavonoids also effectively inhibited basic fibroblast growth factor (bFGF) stimulated growth of BBCE endothelial cells (luteolin: IC_50_ = 1.9 µM), and bFGF or vascular endothelial growth factor (VEGF) induced *in vitro angiogenesis* of cells bovine microvascular endothelial (BME) cells. Thus, luteolin and some other flavonoids might be able to stop the development of solid tumors by suppressing angiogenesis. This, of course, has to be confirmed by *in vivo* studies with adequate concentrations.

Kawaii *et al.* [13] tested 27 flavonoids from *Citrus species* for antiproliferative activity on several cancer and normal human cell lines, using an assay with Alamar Blue, an oxidation-reduction indicator. All flavonoids displayed antiproliferative effects in normal human cell lines at about 40 µM, but only a few were effective at lower concentrations in cancer cell lines. Luteolin was the strongest inhibitor with IC_50_ of 3.1 µM in A 549 human lung carcinoma cells, 2.3 µM in mouse B16 melanoma 4A5 cells, 2.0 µM in human T-cell leukemia cells CCRF-HSB-2, and 1.3 µM in human gastric cancer TGBC11TKB cells. Only natsuidaidain was similarly effective, followed by quercetin and tangeritin. 

Luteolin and 4,4’-dihydrochalcone were the strongest inhibitors of human leukemic CEM-1 and CEM-C7 cell proliferation among seven dietary substances selected. Incubation with 30 µM of either compound for 24 h resulted in complete ATP depletion and inhibition of glucose uptake [14]. These results are confirmed by experiments on leukemic lymphocytes (P 388 from mouse) where luteolin had the lowest IC_50_ value (1µM) of all flavonoids tested [15]. 

Cherng *et al.* [16] determined the inhibitory effect of 10 minor dietary constituents (e.g. baicalein, linalool, caffeic acid, ferulic acid) on proliferation in human cancer cell lines from 10 different organs, and in normal leucocytes. Luteolin was effective in all 10 cancer types, with lowest IC_50_ for carcinoma of the stomach (25 µM), cervix (27 µM), lung (41 µM) and bladder (68 µM). Baicalein had a narrower spectrum, but was partly more specific (ratio of inhibitory concentration in cancer to normal cells). Most other compounds were ineffective, but linalool, a monoterpene, had a low IC_50_ of 1 µM and extremely high specificity in cervix cancer cells.

Takahashi *et al.* [17] found luteolin and apigenin to strongly inhibit growth of HL60 human leukemia cells and to induce morphological differentiation into granulocytes, while quercetin, naringenin, galangin and kaempferol also caused inhibition but not morphological differentiation. Growth-inhibition by luteolin was seen at about 50 µM and above, differentiation at 100 µM. Ko *et al.* [18] determined for luteolin an IC_50_ of 15 µM for inhibition of proliferation in HL60 cells. Chang *et al.* [19] tested 23 different flavonoids in HL60 cells, and found luteolin to be second-best with an IC_50_ of 12.5 µM, inferior to 3,6-dihdroxyflavone only (8.8 µM). Other flavonoids, however, were also fairly effective. Luteolin (IC_50_: 19 µM) was the strongest inhibitor of 8 selected flavonoids on human A431 squamous cell cancer cells, closely followed by quercetin (21 µM) [20]. In lung carcinoma cells, Kim *et al.* [21] found inhibition of proliferation with IC_50_ = 12 µM.

Knowles *et al.* [22] compared the effects of selected bioflavonoids on the proliferation of androgen-independent human prostatic cancer cells (PC-3). Complete growth retardation was observed in PC-3 cells treated with 100 µM quercetin, kaempferol, and luteolin, while isomolar concentrations of genistein, apigenin, and myricetin suppressed PC-3 proliferation by 73%, 70%, and 59%, respectively (p < 0.05). Naringenin and rutin were not as effective and inhibited growth by < 25%. Apoptosis was not evident, even when concentrations of quercetin or kaempferol were raised to 100 µM. A block in G2/M phase progression was observed and seemed to contribute significantly to the antiproliferative effect. Lim *et al.* [23] also reported cell cycle arrest in G2/M by downregulation of cyclin B1 expression and of cell division cycle activity and apoptosis in HT-29 human colon cancer cells at concentrations from 20 to 60 µM. Plaumann *et al.* [24] found tumor growth inhibition and G2/M cell cycle arrest as well as apoptosis induced by 53 µM luteolin to depend on p53 tumor suppressor gene, since p53 knockout fibroblasts were not sensitive to luteolin. Chang *et al.* [25] observed apoptosis and cell cycle arrest in G0/G1 phase in five human hepatoma cell lines, with IC_50_ = 12.5 µM for luteolin in HL60 cells. Cell cycle arrest in S-phase was found in CH27 human lung squamous carcinoma cells with 50 µM luteolin, while anti-oxidant enzymes such as CAT and SOD increased [26]. 

Luteolin was also isolated from two Asian plants traditionally used as anticancer medicines, *Epimedium koreaonum*, and *Terminalia arjuna*, and found to inhibit proliferation of MCF-7 (breast cancer) and HepG2 (liver cancer) cells in a dose-dependent manner [27]; [28]. 

Human lung squamous carcinoma CH27 cells underwent apoptosis upon stimulation with 50 µM luteolin. An increase of anti-oxidant enzymes as CAT and SOD as observed, but not the production of reactive oxygen species and disruption of mitochondrial membrane potential. Therefore, the effects of luteolin on CH27 cell apoptosis were suspected to result from the antioxidant rather than the pro-oxidant action of luteolin [29]. Luteolin inhibits DNA-synthesis induced by thrombocyte growth factor in mesangial cells from mouse kidney and thereby stops proliferation. The IC_50_ values were 1.5 μM for luteolin, 1.8 μM for rosmarinic acid, and 6.1 μM for luteolin-7-O-glucoside [30]. Luteolin was one of the most effective of 29 flavonoids tested for cytotoxicity in HeLa cells (uterine cancer) [31]. In the microculture tetrazolium (MTT) assay, luteolin displayed strong cytotoxic activity in human cancer cell lines. Upon continuous incubation, luteolin inhibited growth of GLC4 (lung cancer) cells with IC_50_ of 40.9 µM and COLO 320 (colon cancer) cells with IC_50_ of 32.5 µM. Luteolin-7-O-glucoside (cynaroside) and some other flavones gave similarly results, whereas flavonols were less effective. However, the reference substance cisplatin and the arnica-derived sesquiterpene lactone helenalin displayed cytotoxicity at concentrations of about 1 µM and below [32]. 

Despite all these promising results it has to be considered that cell lines have been used avoiding any biotransformation. Therefore, final conclusion can only be drawn from *in vivo* studies.

#### 3.1.2 *In vivo*

Growth of Lewis lung cancer cells implanted on the flank of mice was inhibited by 40% with 2 mg/kg luteolin and by 60% with 10 mg/kg [21]. Luteolin (5 to 50 mg/kg b.w. i.p.) inhibited tumor growth in nude mice with xenografted SKOV3.ip1 (human ovarian cancer cell line) induced tumors [33]. Selvendiran *et al.* [34] supplied nude mice with xenografted HAK-1B hepatoma tumors with food containing 50 or 200 ppm luteolin, resulting in a highly significant, dose-dependent reduction of mean tumor volume from about 1200 mm^3^ to 300 mm^3^.

Several *in vivo* investigations demonstrated suppression of tumor in prostate cancer, e.g. in immunodeficient SCID mice with androgen-sensitive human prostate adenocarcinoma LNCaP cells xenograft tumor growth. Male mice receiving 25 to 50 mg/kg luteolin i.p. daily had significantly reduced serum PSA levels and only 25 to 50 % of the tumor volumes of controls [35]. Fang *et al.* [36] determined the effects of luteolin on prostate cancer PC-3 xenograft in nude mice. Tumor weight after 18 days was 180 mg in controls, 125 mg with 5 mg/kg and 110 mg with 10 mg/kg i.p. luteolin (p = 0.011). There was no indication of systemic toxicity to mice as evidenced by normal food intake and body weight. More apoptotic cells were found in tumors treated with luteolin than in those treated with solvent in the Tunnel assay. Manju *et al.* [37] studied the chemopreventive effect of luteolin on bacterial enzymes in a colon carcinogenesis model induced by dimethylhydrazine (DMH) (20 mg/kg), administered subcutaneously once a week for the first 15 weeks and then discontinued. Colon cancer incidence and the activities of bacterial enzymes β-glucuronidase (in the proximal colon, distal colon, intestines, liver, and colon contents) and mucinase (colon and fecal contents) were significantly increased in DMH-treated rats at the end of 30 weeks. On luteolin administration (0.1, 0.2, or 0.3 mg/kg p.o everyday.), colon cancer incidence, number of tumors per rat and tumor size were reduced by 70 to 90 % both in initiation and post-initiation stages of colon carcinogenesis in a dose-dependent manner; the activities of beta-glucuronidase and mucinase in colon bacteria were significantly decreased. An increase in β-glucuronidase activity may augment the hydrolysis of glucuronide conjugates, liberating toxins, while an increase in the mucinase activity may enhance hydrolysis of protective mucins in the colon. Thus luteolin exerts chemopreventive and anticarcinogenic effects against DMH induced colon cancer. Markaverich *et al.* [38] demonstrated inhibition of growth by luteolin in normal and in cancerous prostate cells in mice. Prostate weight was reduced by 60% within 2 weeks at a daily dose of 20 mg/kg p.o. without adverse effects on total body weight, seminal vesicles or testes. Proliferation of both LNCaP and PC-3 prostate cancer cell lines was inhibited *in vitro* in a concentration-dependent manner from about 3 µM to complete inhibition at 30 µM. Growth of PC-3 xenografts to nude mice was significantly delayed by adding 250 µg/mL to the animals’ water supply. Blocking of type II [3H]-estradiol receptors was found as underlying mechanism. Resident macrophages control metastatic spread of colon carcinoma cells in liver and peritoneal tumor models. However, it was proposed that newly recruited macrophages develop into M2 macrophages which induce tumor progression by stimulating angiogenesis and proliferation, as they are exposed to a microenvironment that favors alternative activation. Monocyte migration was diminished after treatment with rutin and luteolin in an experimental autoimmune encephalomyelitis animal model, and increased tumor development was observed. Immunohistochemical analyses showed that the number of ED2(+) resident macrophages was normal in tumors of animals that received rutin and luteolin treatment. However, the number of ED1(+) cells (marker immature macrophages) was reduced, indicating decreased macrophage recruitment. Thus, inhibition of monocyte migration promotes tumor growth, supporting that not only resident, but also newly recruited macrophages limit peritoneal colon carcinoma metastases development [39]. However, only these special tumor-associated macrophages in a special stroma environment are involved in tumor proliferation, whereas classically activated macrophages have cytotoxic effects and produce inflammatory cytokines that inhibit tumor growth. To define the general effect and mechanism of rutin and luteolin on tumor-associated macrophages more experiments are required. 

Effects of *Perilla* leaf extract (PLE) and luteolin, which is regarded as the main active component of PLE, on dimethylbenz-[a]-anthrazene (DMBA) and tissue plasminogen activator (TPA) induced skin papillomas in mice were investigated [40]. Topical application of PLE prior to TPA treatment in DMBA initiated mouse skin resulted in a significant reduction in tumor incidence and multiplicity. An even more potent preventive effect was observed with topical application of 1 µg luteolin in 0.2 ml of acetone per mouse and application. Luteolin reduced the mean number of tumors developing within 2 weeks after induction from 6 to only 1 per mouse. When PLE was dissolved in drinking water at a 0.05% dose and mice ingested it ad libitum, no significant difference was observed in tumor incidence or multiplicity but in tumor volume between the PLE-treated and untreated groups.

### 3.2 Anti-carcinogenic mechanisms

#### 3.2.1 Protection from carcinogenic agents

Like other flavonoids, luteolin can strongly absorb UV light, and derivatives may be used in sun protection products for prevention of skin cancer and early skin ageing [41,42].

Protection of DNA from oxidative stress was demonstrated *in vitro* in the COMET assay [43,44,45,46,47] and *in vivo* with gamma-ray irradiated mice [48].

Luteolin also induces expression of antoxidant enzymes, as shown by Sharma *et al.* [49] in normal astrocytes. Samy *et al.* [50] treated female Wistar rats with luteolin (30 mg/kg, p.o.), combined with cyclophosphamide (10 mg/kg, i.p.) (luteolin+CYC) administered for 20 days; and CYC individually for 10 days against DMBA induced mammary carcinogenesis in Wistar rats. CYC alone had the highest potential in reducing tumor number and volumes, but the authors state that long term treatment is toxic to the animals as seen in a strong decrease of body weight. Combination treatment of CYC and luteolin, which has a lower anti-tumor potential, eliminates the toxic effect. Levels and activities of superoxide dismutase (SOD), catalase (CAT) and glutathione peroxidase (GPx) in liver, kidney and breast were reduced by 50 to 80% in mice with induced breast cancer, but were re-established to normal by the combination treatment. Unfortunately, there are numerous contradictions between text, tables and figures in this paper. Manju *et al.* [51] observed enhancement of plasma and hepatic anti-oxidant status (glutathione GSH, pyrogallol peroxidase PPx, glutathione-S-transferase GST, glutathione reductase GR, SOD, CAT, Vitamin C, Vitamin A and β-carotene) in rats with DMH induced colon cancer upon intragastric administration of 0.2 mg/kg luteolin.

Choi *et al.* [52] screened 27 flavonoids for antimutagenic activity against aflatoxin B1(AFB1) and *N*-methyl-*N'*-nitro-*N*-nitrosoguanidine (MNNG) in *Salmonella typhimurium* TA100 in the Ames test. In mixed applications of AFB1 (1 µg/plate) with flavonoids (300 µg/plate) in the presence of a mammalian metabolic activation system (S9 mix), chrysin, apigenin, luteolin and its glucoside, kaempferol, fisetin, morin, naringenin, hesperetin, persicogenin, (+)-catechin and (-)-epicatechin reduced mutation rates caused by AFB1 by more than 70%. Anti-mutagenic capacity AFB1 against seemed to depend on free 5-,7-hydroxyl groups, while saturation of the 2,3-double bond or elimination of the 4-keto group did not affect activity. Little or no anti-mutagenic activity against MNNG (0.5 µg/plate) was observed except for flavone.

Taj *et al.* [53] found chemopreventive activity of quercetin and luteolin against chromosomal alterations of long-term feeding on deep-fried fish and mutton in rat bone marrow cells. Groups of rats were treated with flavonoids through pre-, simultaneous- and post-treatment regimens, and bone marrow was analysed for presence of micronuclei and chromosome aberrations. Pre-treatment was most effective in inhibition of mutagenicity at every dose tested. Luteolin was a better protective agent than quercetin. It suppressed 93% of genetic damage in the micronucleus assay and 95% of chromosome aberrations induced by fish extract (p < 0.001 in both groups). Mutton extract-induced micronuclei and chromosome aberrations were reduced by 85% and 90%, respectively, by luteolin and by 79% and 76%, respectively, by quercetin. 

Luteolin in concentrations from 4 to 400 µM inhibited N-acetyltransferase (NAT) in human HL-60 and mouse L 1210 leukemia cells and decreased DNA-2-aminofluorene adduct formation in a non-competitive, dose-dependent manner with IC_50_ of 40 µM [54] in the same way as does paclitaxel [55].

This was also found in human J5 liver tumor cells [56], and in human T24 bladder cancer cells with accompanying inhibition of NAT1 mRNA gene expression [57]. Inhibition lasted for 24 – 48 h in intact cancer cells.

#### 3.2.2 Inhibition of cell adhesion and invasion

Clinical observations suggest that hepatocyte growth factor HGF (known as scatter factor, SF) and its receptor, the c-Met tyrosine kinase, can promote metastasis of hepatoma cells while stimulating tumor invasiveness. Lee *et al.* [58] investigated the effect of flavonoids including luteolin, quercetin, baicalein, genistein, taxifolin and catechin on HGF-mediated migration and invasion of HepG2 cells. Luteolin presented the highest potential on anti-migration and anti-invasion determined by Boyden chamber assay with significant inhibition from 5 µM up to complete inhibition at 40 µM. Furthermore, luteolin inhibited HGF-induced cell scattering and cytoskeleton change such as filopodia and lamellipodia. Luteolin suppressed the phosphorylation of c-Met, the membrane receptor of HGF, as well as ERK1/2 and Akt, but not JNK1/2, which is activated by HGF. Luteolin (40 µM) effect on HGF-induced HepG2 cell invasion was stronger than that of 50 µM PD98059, a specific inhibitor of MEK, an upstream kinase regulating ERK1/2, and similar to that of 200 µM wortmannin, a PI-3-K inhibitor. 

Lansky *et al.* [59] tested compounds with known anti-cancer effects from pomegranate fruit (*Punica granatum*), as potential inhibitors of *in vitro* invasion of human PC-3 prostate cancer cells in an assay employing Matrigel artificial membranes. Ellagic acid (E), caffeic acid (C), luteolin and punicic acid (P) significantly inhibited invasion when employed individually at a concentration of 4 µg/mL. When C, P, and luteolin were equally combined at the same gross dosage as when the compounds were tested individually, a superadditive inhibition of invasion was observed. 

In the human epithelial cell cancer line A431, luteolin (IC_50_ = 19 µM) and quercetin (IC_50_ = 21 µM) were the most potent of eight flavonoids to inhibit cell proliferation and secretion of matrix metalloproteinases MMP-2 and MMP-9, two gelatinases involved in metastasis. Luteolin reduced activity of MMP2 by 73% and of MMP 9 by 94% [20]. 

#### 3.2.3 Topoisomerase inhibition

Luteolin inhibits topoisomerase I and II, thus inhibiting cell replication and DNA repair and promoting cell death of apoptosis. Yamashita *et al.* [60] demonstrated that this process is differentially affected by specific flavonoids. To clarify the mechanism underlying the carcinogenic effects of quercetin, they compared DNA damage occurring during apoptosis induced by quercetin with that occuring during apoptosis induced by luteolin. Both quercetin and luteolin similarly induced DNA cleavage with subsequent DNA ladder formation, characteristics of apoptosis, in HL-60 cells. In HP 100 cells, an H_2_O_2_-resistant clone of HL-60 cells, the extent of DNA cleavage and DNA ladder formation induced by quercetin was less than that in HL-60 cells, whereas differences between the two cell types were minimal after treatment with luteolin. In addition, quercetin increased the formation of 8-oxodG, an indicator of oxidative DNA damage, in HL-60 cells but not in HP 100 cells. Luteolin did not increase 8-oxodG formation, but inhibited topoisomerase II activity of nuclear extract more strongly than quercetin, and cleaved DNA by forming a luteolin-topo II-DNA ternary complex. These results suggest that quercetin induces H_2_O_2_-mediated DNA damage, resulting in apoptosis or mutations, whereas luteolin induces apoptosis via topoisomerase II-mediated DNA cleavage. Topoisomerase II inhibition was also seen in Chinese hamster ovary cells AA8 by Cantero *et al.* [61], at concentrations of 30 – 80 µM, where also very high yields of metaphases with diplochromosomes appeared. The authors discuss a possible carcinogenic effect, as was discussed for various flavonoids before [62], [63], and question the usefulness of these substances in cancer therapy. Luteolin completely inhibited catalytic activity of isolated rat liver DNA topoisomerase I at a concentration of 40 µM, with an IC_50_ of 5 µM [64]. Preincubation of enzyme with luteolin before adding a DNA substrate increases inhibition of catalytic activity (IC_50_=0.66 µM). Treatment of DNA with luteolin before addition of topoisomerase I reduces this inhibitory effect. Subsequent fluorescence tests show that luteolin not only interacts directly with the enzyme but also with the substrate DNA, and intercalates at a very high concentration (>250 µM) without binding to the minor groove. Inhibition of topoisomerase I by luteolin is due to stabilization of topoisomerase-I DNA-cleavable complexes [64]. 

#### 3.2.4 PI-3-Kinase / Akt regulation / MAPK / ERK / JNK

Ruiz *et al.* [65] showed that apigenin and luteolin selectively blocked Akt phosphorylation/activity in TNF-α induced murine non-carcinoma intestinal epithelial cell (IEC) line Mode-K with IC_50_ of 20 µM; only luteolin at 100 µM caused long-lasting inhibition over 24 h, too.

Luteolin inhibited insulin-like growth factor -1 (IGF-1)- induced activation of IGF-1R and Akt in prostate cancer PC-3 and DU145 cells [36]. Inhibition of Akt appeared at luteolin concentrations of 10 µm, was almost complete at 40 µM, and resulted in decreased phosphorylation of its downstream targets. Luteolin also inhibited the IGF-1-induced activation of EGFR and MAPK/ERK signaling. Luteolin inhibited expression of cyclin D1 and increased expression of p21. As a result, luteolin suppressed proliferation and induced apoptosis in prostate cancer cells. Agullo *et al.* [66] determined an IC_50_ of 8 µM for PI-3-K inhibition *in vitro*, which is lower than IC_50_ of apigenin, quercetin, and fisetin, but higher than of myricetin (1.8 µM). Almost exactly the same value (8.65) µM was determined for the inhibition of PI-3-K p1 10β human recombinant subunit [67]. Luteolin caused downregulation of ERK and Akt in lung cancer cells [21], and inhibited EGF induced EGFR phosphorylation by 47% in epithelial cancer cells at 20 µM [20]. Shi *et al.* [68] observed a significant increase of p53 protein level in three luteolin-treated cancer cell lines at 20 to 40 µM without increase of p53 mRNA level, indicating the possible effect of luteolin on p53 posttranscriptional regulation. They identified the critical role of c-Jun NH(2)-terminal kinase (JNK) in regulation of p53 protein stability: luteolin activates JNK, and JNK then stabilizes p53 via phosphorylation, leading to reduced ubiquitination and proteasomal degradation. In an *in vivo* nude mice xenograft model 40 mg/kg b.w. luteolin i.p., three times a week, enhanced the cancer therapeutic activity of cisplatin via p53 stabilization and accumulation; mean tumor weight after three weeks was reduced from 1.28 g to 0.46 g. Dose-dependent accumulation of p53 leading to apoptosis and cell cycle arrest after stimulation with luteolin (53 and 110 µM), apigenin and quercetin was also reported by Plaumann [24] for the non-tumor mouse embryo fibroblast cell line C3H10T1/2CL8;the effects of luteolin and apigenin were long-lasting, while quercetin induced p53 accumulation only for 1-2 hours unless supplied continuously.

Overexpression of HER2/neu, a receptor of the EGFR family, confers resistance to various therapeutic regimens in breast cancer and correlates with a poor clinical prognosis. Chiang *et al.* [33] showed that luteolin is a potent stimulator of HER2/neu degradation. Luteolin 40 µM reduced levels of Her2/neu to 35 or 30% of control, depending on the cell line. Cell proliferation and induced apoptosis in HER2-overexpressing cancer cells were significantly reduced by 10 µM luteolin. Low doses of luteolin up-regulated p21 expression and high doses of luteolin down-regulated its expression. Akt/mammalian target of rapamycin (mTOR) signaling was only transiently inhibited by low doses of luteolin, which suggested that the inability to cause sustained Akt/mTOR inhibition may contribute to p21 induction and provide a survival advantage to HER2/neu-overexpressing cancer cells. Combined use of luteolin (5 to 10 µM) and mTOR inhibitor rapamycin (100 nM) prevented low doses of luteolin from inducing p21 expression, and HER2/neu-overexpressing cancer cells would be sensitized toward luteolin-induced apoptosis. Cell viability was reduced by 50%. In addition, p21 small interfering RNA also increased luteolin-induced cell death. In nude mice with xenografted SKOV3.ip1-induced tumors, luteolin significantly inhibited HER2/neu expression and tumor growth in a dose-dependent manner, and rapamycin further enhanced the effect of luteolin with concomitant p21 inhibition. These results reveal an intriguing finding that suppressing p21 expression might have therapeutic implications and further suggest that combination of mTOR inhibitors may be a promising strategy to help increase the efficacy of preventive or therapeutic compounds against HER2/neu-overexpressing tumors. However, this has to be verified under *in vivo* conditions.

Luteolin inhibited proliferation and induced apoptosis in the human prostate cancer cell line LNCaP, and less effectively in the cell lines PC-3 and DU-145. Luteolin simultaneously suppressed intracellular and secreted PSA levels and repressed androgen receptor and heat shock protein 90 expression [35].

Han *et al.* [69] also found androgen receptor antagonism in luteolin and other flavonoids. Ethyl acetate extract from pollen of *Brassica napus* L. showed strong activity in decreasing the secretion of prostate specific antigen (PSA) in LNCaP cells as compared to two other extracts, measured by ELISA with finasteride as positive control. Naringenin; luteolin; kaempferol; kaempferol 3-(3-E-p-coumaroyl-α-l-rhamnopyranoside); and kaempferol 3-(2,3-di-*E*-*p*-coumaroyl-α-l-rhamno-pyranoside) were subsequently isolated from the active extract using bioassay-guided fractionation. All these compounds inhibited PSA secretion significantly, with IC_50_ values in the range of 5-50 µM. Luteolin, kaempferol 3-(3-E-p-coumaroyl-α-l-rhamno-pyranoside) and kaempferol 3-(2,3-di-*E*-*p*-coumaroyl-α-l-rhamno-pyranoside (but not naringenin or kaempferol) showed moderate cytotoxicity to LNCaP cells within the active concentration range. *Brassica napus* L. has been used in China to treat benign prostatic hyperplasia (BPH) for over decades.

#### 3.2.5 Mitochondrial activation of apoptosis

A decrease of anti-apoptotic proteins Bcl-2 and Bcl-XL was observed upon luteolin treatment in various cancer cell lines, as well as an increase of pro-apoptotic members of the Bcl-2 family, Bax and Bak, and their translocation to mitochondria [25]; [70]; [21]; [71]; [23]; [34]. Mitochondrial membrane potential in HL-60 leukemia cells [70] and LLC lung carcinoma cells [21] decreased, and cytochrome C was released into cytosol in these studies, also reported for HepG hepatoma cells [71]. These events were associated with activation of caspase 9 and caspase 3, which was also found in malignant cells but not in normal human peripheral blood mononuclear cells (PBMC) [72]. In rat hepatoma H4IIE cells, Michels *et al.* [73] observed a 6-fold activity of caspase 9 starting after 6 h, while caspase 2 and 3/7 were activated after 12 h to a 4-fold activity level using a high concentration of 250 µM. After 24 h, also caspase 8 /10 were activated. Using relatively high concentrations of luteolin (100 to 250 µM, 24 h incubation), Michels observed oligonucleosomal DNA cleavage. Inactivation of the DNA repair enzyme poly (ADP-ribose) polymerase (PARP) and activation of DNA fragmentation factor DFF-45 were reported at a concentration of 60 µM [70] and 50 µM [26].

Thus, the complete apoptotic process of the survival pathway (PI-3-K/Akt) and MAPK pathway including mitochondrial processes and activation of caspases and downstream enzymes was demonstrated after luteolin application to cancer cell lines or in biochemical assays. 

#### 3.2.6 Death receptor-induced apoptosis and cell cycle arrestment mechanisms

Horinaka *et al.* [72] found, that luteolin not only induced the mitochondrial pathway of apoptosis at a concentration of 5 to 40 µM, but also caspase 8/10 activation and death receptor (DR-5) expression in human malignant cells. Suppression of DR-5 expression with small interfering RNA (siRNA) efficiently reduced caspase activation and apoptosis induced by 20 µM luteolin. On the other hand, luteolin did neither induce DR-5 expression nor apoptosis in normal human PBMC. Further *in vivo* studies must prove whether the authors’ conclusion is justified, that luteolin might be promising for cancer treatment. 

Tumor necrosis factor (TNF)-related apoptosis-inducing ligand (TRAIL) is an important member of the TNF superfamily with great potential in cancer therapy. Pretreatment with a noncytotoxic concentration of 40 µM luteolin significantly sensitized TRAIL-induced apoptosis in both TRAIL sensitive (HeLa) and TRAIL resistant cancer cells (CNE1, HT29, and HepG2) through enhanced caspase 8 activation and caspase 3 maturation [74]. The protein level of X-linked inhibitor of apoptosis protein (XIAP) was markedly reduced in cells treated with luteolin and TRAIL, and ectopic expression of XIAP protected against cell death induced by luteolin and TRAIL, showing that luteolin sensitizes TRAIL induced apoptosis through down-regulation of XIAP. Luteolin and TRAIL promoted XIAP ubiquitination and proteasomal degradation. Protein kinase C (PKC) activation prevented cell death induced by luteolin and TRAIL via suppression of XIAP down-regulation. Moreover, luteolin inhibited PKC activity, and bisindolylmaleimide I, a general PKC inhibitor, simulated luteolin in sensitizing TRAIL induced apoptosis. 

Selvendiran *et al.* [34] investigated the effect of luteolin on signal transducer and activator of transcription 3 (STAT3)/Fas signaling. A clear induction of apoptosis was found in luteolin-treated HLF hepatoma cells in a time- and dosage-dependent manner from 5 to 50 µM. In concert with caspase 8 activation by luteolin, an enhanced expression in functional Fas/CD95 was identified. Consistent with the increased Fas/CD95 expression, a drastic decrease in the Tyr(705) phosphorylation of STAT3, a known negative regulator of Fas/CD95 transcription, was found within 20 minutes in the luteolin-treated cells, leading to down-regulation in the target gene products of STAT3, such as cyclin D1, survivin, and vascular endothelial growth factor. 

Lim *et al.* [23] examined luteolin mediated regulation of cell cycle progression and apoptosis in the HT-29 human colon cancer cell line. Luteolin decreased DNA synthesis and viable HT-29in a concentration-dependent manner from 20 µM to 60 µM. It inhibited cyclin-dependent kinase (CDK) 4 and 2 activity, resulting in G1 arrest with a concomitant decrease of phosphorylation of retinoblastoma protein. Activities of CDK4 and CDK2 decreased within 2 h after luteolin treatment, with a 38% decrease in CDK2 activity (P < 0.05) observed in cells treated with 40 µM luteolin. Luteolin (60 µM) inhibited CDK2 activity in a cell-free system, suggesting that it directly inhibits CDK2. Cyclin D1 levels decreased after luteolin treatment, although no changes in expression of cyclin A, cyclin E, CDK4 or CDK2 were detected. Luteolin also promoted G2/M arrest at 24 h post treatment by downregulating cyclin B1 expression and inhibiting cell division cycle (CDC) 2 activity. Luteolin decreased expression of p21^CIP1/WAF1^ and survivin. These data demonstrate that the extrinsic pathway of apoptosis induction by luteolin is closely linked to inhibition of specific regulators of cell cycle progression.

#### 3.2.7 Inhibition of fatty acid synthase (FASN)

Expression and activity of Fatty Acid Synthase (FASN; the sole enzyme capable of the reductive de novo synthesis of long-chain fatty acids from acetyl-CoA, malonyl-CoA, and nicotinamide adenine dinucleotide phosphate -NADPH-) are extremely low in nearly all nonmalignant adult tissues, whereas FASN is significantly up-regulated or activated in many cancer types. *In vitro* and *in vivo* studies have confirmed the potential of FASN as a target for anti-neoplastic intervention. FASN inhibitors such as the mycotoxin cerulenin, the cerulenin derivative C75, the β-lactone orlistat, the green tea polyphenol epigallocatechin-3-gallate (EGCG) and other naturally occurring flavonoids (i.e., luteolin, quercetin, and kaempferol), as well as the antibiotic triclosan, have been shown to limit cancer cell growth by inducing apoptotic cell death [75].

#### 3.2.8 Sensitization to chemotherapy

Interestingly, flavonoids may increase susceptibility of cancer cells to chemotherapy [76]. Over-expression of glutathione-conjugate transporting multidrug-resistance proteins (MRP) is supposed to counteract the effects of anti-carcinogenic drugs. A number of flavonoids inhibited MRP-1 in the human MCF7 breast cancer cell line effectively; a C2-C3 double-bond and 3',4'-dihydroxilation are structural requirements for strong inhibition. Luteolin had an IC_50_ of only 0.8 µM and was the most effective flavonoid tested, followed by quercetin with 1.3 µM, concentrations which may be achieved by food supplementation as was shown for quercetin [77].

## 4. Epidemiological evidence for health protective effects of dietary flavone intake

New instruments were developed during the last decades which now allow the estimation of total flavonoid contents in diets, and the most important flavonoid subclasses: the USDA database lists flavones, flavonols, flavanones, flavan-3-ols, anthocyanidins, as well as isoflavones and proantho- cyanidins, and a number of important single components [78]. Investigations in different populations have revealed that mean total flavonoid intake is usually in the order of 100 - 200 mg/d, flavonoles (with quercetin and kaempferol as main components) contribute 5 - 20 mg/d, flavones only about 1 mg, often less; up to 90 % of the flavone fraction may be apigenin, leaving about 0.1 mg/d for luteolin. Considerable concentrations of luteolin are found in some spices like thyme, parsley, sage, and in wild carrots. Celery, spinach, some varieties of peppers and lettuce are our major nutritional luteolin sources. Expectations to find health effects of flavone (or even luteolin) intake by epidemiological correlations should be moderate. It is surprising, after all, that there are indeed some promising new data for these substances. 

In four recent investigations, high flavone intake was always significantly correlated with a lower risk of breast cancer, while results for other flavonoid subclasses were inconsistent. No differentiation was made between the two major flavone components, apigenin and luteolin.

Bosetti *et al.* [79] compared 2,569 Italian women with incident, histologically confirmed breast cancer to 2,588 hospital controls. After allowance for major confounding factors and energy intake, a reduced risk of breast cancer was found for increasing intake of flavones (odds ratio (OR): 0.81, p = 0.02), and flavonols (OR: 0.80; p = 0.06). No significant association was found for other flavonoid subgroups. In a Greek case-control study Peterson *et al.* [80] compared 820 women with breast cancer to 1,548 control women and found a strong, statistically significant inverse association of breast cancer with flavones, but not other flavonoids; a difference in daily flavone intake of 0.5 mg corresponded to a 13 % lower risk. Fink *et al.* [81] investigated a population-based cohort of 1,434 breast cancer patients and 1,440 controls in a case-control study in Long Island, New York, USA. A decrease in breast cancer risk was observed, most pronounced among postmenopausal women for high intake of flavonols (OR: 0.54), flavones (OR 0.61: (0.45, 0.83)), flavan-3-ols (OR: 0.74), and lignans (OR: 0.69). In the same cohort, women ages 25 to 98 years with newly diagnosed first primary invasive breast cancer (n = 1210) were followed for vital status for 6 years [82]. Reduced hazard ratios for all-cause mortality were observed among premenopausal and postmenopausal women for the highest quintile of intake, compared with the lowest, for flavones [0.63 (0.41-0.96)], isoflavones [0.52 (0.33-0.82)], and anthocyanidins [0.64 (0.42-0.98)]. Results were similar for breast cancer–specific mortality. 

While several investigations concerning laryngeal cancer, pharyngeal cancer, stomach cancer, colorectal cancer, prostate cancer or bladder cancer could not detect protective effects of flavones, there are also some significant correlations reported for colorectal cancer [83] and renal cell cancer [84] in Italy. Garcia-Closas *et al.* [85] could not find a correlation between luteolin intake and lung cancer risk; however, this case control study had only 103 cases and could neither confirm a protective effect of total flavonoids, which has been demonstrated consistently in six cohort and four case-control studies [86]. Gates *et al.* [87] analyzed the association between intake of 5 common dietary flavonoids and incidence of epithelial ovarian cancer among 66,940 women in the long time, prospective US Nurses' Health Study. They calculated each participant's intake of myricetin, kaempferol, quercetin, luteolin and apigenin from dietary data collected at multiple time points. The analysis included 347 cases diagnosed between 1984 and 2002, and 950,347 person-years of follow-up. There was no clear association between incidence of ovarian cancer and total intake of the five flavonoids examined, but a significant 40% decrease for kaempferol and a significant 34% decrease for luteolin (OR: 0.66 (0.49-0.91) p-trend = 0.01). If confirmed, these results would provide an important target for ovarian cancer prevention.

## 5. Conclusions

Luteolin can suppress proliferation of various kinds of tumor cells *in vitro* with IC_50_ from about 3 to 50 µM, and inhibited tumor growth effectively *in vivo* when administered, e.g., in concentrations of 50 to 200 ppm in food [34], intragastric application of 0.1 -0.3 mg/kg/d [37] or 5 to 10 mg/kg i.p. [36]; [33]. Whether concentrations effective in these experiments can be reached by supplementation in humans has to be elucidated. Flavonoid pharmacokinetics is complex, since they are usually contained as glucosides in fruits and vegetables, cleaved and glucuronated during uptake. Glucuronides may be metabolized, or stored or set free as aglycones by tissue-specific glucuronidases; thus, plasma concentration may not always be a good measure of bioavailability. 

**Figure 2:**
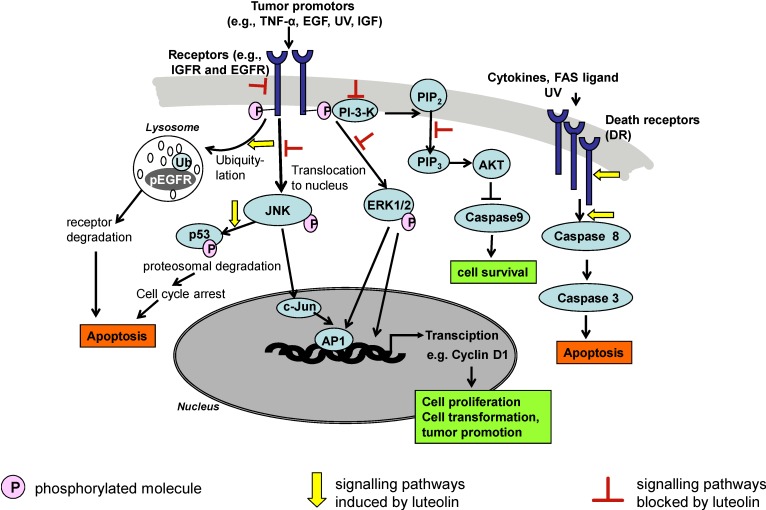
Some signal transduction pathways and their modulation by luteolin. This illustration represents general pathways suggested in the scientific literature and is not to be considered comprehensive or definitive.

The interaction of luteolin with carcinogenic mechanisms is complex and resembles EGCG, a green tea polyphenol, which also has multiple inhibitory effects on tumor induction and promotion, which are partly the same as with luteolin [11]. Figure 2 gives an overview of mechanisms by which luteolin modulates signal transduction pathways relevant for cancer development. 

Although luteolin does not have the pronounced pro-oxidative capacity of some flavonols like quercetin, which may be harmful as well as protective to biological structures depending on conditions, the generally positive safety profile of luteolin observed in pharmacological and toxicological investigations needs further clinical support before recommendations for dietary supplementation in very high amounts can be given. However, luteolin appears as a promising molecule for development of systemic as well as topical cancer treatments. 

Prospective clinical studies would be desirable, but are also difficult, expensive and long-lasting in the field of cancer. There is good epidemiological evidence suggesting that the flavone subclass of the flavonoids (apigenin and luteolin) contributes to the cancer-protective effect of fruits and vegetable food. However, a broader set of epidemiological data for flavones only exists with breast cancer. The differentiation between luteolin and apigenin needs further investigation. Epidemiological studies with even more refined analyses of flavonoid composition on one hand, and more *in vivo* studies of luteolin effects on the development of different cancer types on the other hand appear as the most promising fields for research in the near future.

## References

[1] Block G., Patterson B., Subar A. (1992). Fruit, vegetables, and cancer prevention: a review of the epidemiological evidence. Nutr. Cancer.

[2] Huang D., Ou B., Prior R.L. (2005). The chemistry behind antioxidant capacity assays. J. Agric. Food. Chem..

[3] Soobrattee M.A., Neergheen V.S., Luximon-Ramma A., Aruoma O.I., Bahorun T. (2005). Phenolics as potential antioxidant therapeutic agents: mechanism and actions. Mutat. Res..

[4] Seelinger G., Merfort I., Schempp C.M. (2008). Anti-oxidant, anti-inflammatory and anti-allergic activities of luteolin, a flavone from the Dyer’s weld *Reseda luteola* L.. Planta Med..

[5] Brown J.E., Khodr H., Hider R.C., Rice-Evans C.A. (1998). Structural dependence of flavonoid interactions with Cu^++^ ions: implications for their anti-oxidant properties. Biochem. J..

[6] Heilmann J., Merfort I. (1998). Aktueller Kenntnisstand zum Metabolismus von Flavonoiden. II. Resorption und Metabolismus von Flavonen, Flavanonen, Flavanen, Procyanidinen und Isoflavonoiden. Pharm. Uns. Zeit.

[7] Wittemer S.M., Ploch M., Windeck T., Müller S.C., Drewelow B., Derendorf H., Veit M. (2005). Bioavailability and pharmacokinetics of caffeoylquinic acids and flavonoids after oral administration of Artichoke leaf extracts in humans. Phytomedicine.

[8] Boersma MG., van der Woude H., Bogaards J., Boeren S., Vervoort J., Cnubben N.H., van Iersel M.L., van Bladeren P.J., Rietjens I.M. (2000). Regioselectivity of phase II metabolism of luteolin and quercetin by UDP-glucuronosyl transferases. Chem. Res. Toxicol..

[9] Shimoi K., Nakayama T. (2005). Glucuronidase deconjugation in inflammation. Methods Enzymol..

[10] Merfort I., Heilmann J, Hagedorn-Leweke U., Lippold B.C. (1994). *In vivo* skin penetration studies on chamomile flavones. Pharmazie.

[11] Chen L., Zhuang H.-Y. (2007). Cancer preventive effects of the green tea polyphenol (-)-epigallocatechin-3-gallate. Molecules.

[12] Fotsis T., Pepper M.S., Aktas E., Breit S., Rasku S., Adlercreutz H., Wähälä K., Montesano R., Schweigerer L. (1997). Flavonoids, dietary-derived inhibitors of cell proliferation and *in vitro* angiogenesis. Cancer Res..

[13] Kawaii S., Tomono Y., Katase E., Ogawa K., Yano M. (1999). Antiproliferative activity of flavonoids on several cancer cell lines. Biosci. Biotechnol. Biochem..

[14] Post J.F., Varma R.S. (1992). Growth inhibitory effects of bioflavonoids and related compounds on human leukemic CEM-C1 and CEM-Cz cells. Cancer Lett..

[15] Hirobe Ch., Quiao Z.-S., Takeya K., Itokawa H. (1997). Cytotoxic flavonoids from *Vitex agnus castus*. Phytochemistry.

[16] Cherng J.M., Shieh D.E., Chiang W., Chang M.Y., Chiang L.C. (2007). Chemopreventive effects of minor dietary constituents in common foods on human cancer cells. Biosci. Biotechnol. Biochem..

[17] Takahashi T., Kobori M., Shinmoto H., Tsushida T. (1998). Structure-activity relationships of flavonoids and the induction of granulocytic- or monocytic-differentiation in HL60 human myeloid leukemia cells. Biosci. Biotechnol. Biochem..

[18] Ko W.G., Kang T.H., Lee S.J., Kim Y.C., Lee B.H. (2002). Effects of luteolin on the inhibition of proliferation and induction of apoptosis in human myeloid leukaemia cells. Phytother. Res..

[19] Chang H., Mi M.T., Gu Y.Y., Yuan J.L., Ling W.H., Lin H. (2007). Effects of flavonoids with different structures on proliferation of leukemia cell line HL-60 [Article in Chinese]. Ai Zheng.

[20] Huang Y.T., Hwang J.J., Lee P.P., Ke F.C., Huang J.H., Kandaswami D., Middleton E., Lee M.T. (1999). Effects of luteolin and quercetin, inhibitors of tyrosine kinase, on cell growth and metastasis-associated properties in A 431 cells overexpressing epidermal growth factor receptor. Br. J. Pharmacol..

[21] Kim J.H., Lee E.O., Lee H.J., Ku J.S., Lee M.H., Yang D.C., Kim S.H. (2007). Caspase activation and extracellular signal-regulated kinase/Akt inhibition were involved in luteolin-induced apoptosis in Lewis lung carcinoma cells. Ann. N. Y. Acad. Sci..

[22] Knowles L.M., Zigrossi D.A., Tauber R.A., Hightower C., Milner J.A. (2000). Flavonoids suppress androgen-independent human prostate tumor proliferation. Nutr Cancer.

[23] Lim D.Y., Jeong Y., Tyner A.L., Jung H.Y.P. (2007). Induction of cell cycle arrest and apoptosis in HT-29 human colon cancer cells by the dietary compound luteolin. Am. J. Physiol. Gastrointest. Liver Physiol..

[24] Plaumann B., Fritsche M., Rimpler H., Brandner G., Hess R.D. (1996). Flavonoids activate wild-type p53. Oncogene.

[25] Chang J., Hsu Y., Kuo P., Kuo Y., Chiang L., Lin C. (2005). Increase of Bax/ Bcl-XL ratio and arrest of cell cycle by luteolin in immortalized human hepatoma cell line. Life Sci..

[26] Leung H.W, Wu C.H., Lin C.H., Lee H.Z. (2005). Luteolin induced DNA damage leading to human lung squamous carcinoma CH27 cell apoptosis. Eur. J. Pharmacol..

[27] Wang T., Zhang J.C., Chen Y., Huang F., Yang M.S., Xiao P.G. (2007). Comparison of antioxidative and antitumor activities of six flavonoids from Epimedium koreanum [Article in Chinese]. Zhongguo Zhong Yao Za Zhi.

[28] Pettit G.R., Hoard M.S., Doubek D.L., Schmidt J.M., Pettit R.K., Tackett L.P., Chapuis J-Ch. (1996). The cancer cell growth inhibitory constituents of Terminalia arjuna (Combretaceae). J. Ethnopharmacol..

[29] Leung H.W., Kuo C.L., Yang W.H., Lin C.H., Lee H.Z. (2006). Antioxidant enzymes activity involvement in luteolin-induced human lung squamous carcinoma CH27 cell apoptosis. Eur. J. Pharmacol..

[30] Makino T., Ono T., Muso E., Honsa G. (1998). Inhibitory effect of Perilla frutescens and its phenolic constituents on cultured murine mesangial cell proliferation. Planta Med..

[31] Mori A., Nishino Ch., Enoki N., Tawata S. (1988). Cytotoxicity of plant flavonoids against HeLa cells. Phytochemistry.

[32] Woerdenbag H., Merfort I., Passreiter C.M., Schmidt Th.J., Willuhn G., van Uden W., Pras N., Kampinga H.H., Konings A.W. (1994). Cytotoxicity of flavonoids and sesquiterpene lactones from arnica species against the GLC4 and the COLO 320 cell lines. Planta Med..

[33] Chiang C.T., Way T.D., Lin J.K. (2007). Sensitizing HER2-overexpressing cancer cells to luteolin-induced apoptosis through suppressing p21^(WAF1/CIP1)^ expression with rapamycin. Mol. Cancer. Ther..

[34] Selvendiran K., Koga H., Ueno T., Yoshida T., Maeyama M., Torimura T., Yano H., Kojiro M., Sata M. (2006). Luteolin promotes degradation in signal transducer and activator of transcription 3 in human hepatoma cells: an implication for the antitumor potential of flavonoids. Cancer Res..

[35] Chiu F.L., Lin J.K. (2008). Downregulation of androgen receptor expression by luteolin causes inhibition of cell proliferation and induction of apoptosis in human prostate cancer cells and xenografts. Prostate.

[36] Fang J., Zhou Q., Shi X.L., Jiang B.H. (2007). Luteolin inhibits insulin-like growth factor 1 receptor signaling in prostate cancer cells. Carcinogenesis.

[37] Manju V., Nalini N. (2007). Protective role of luteolin in 1,2-dimethylhydrazine induced experimental colon carcinogenesis. Cell Biochem. Funct..

[38] Markaverich B.M., Alejandro M.A. (1997). Bioflavonoids, type II [^3^H] estradiol binding sites and prostatic cancer cell proliferation. Int. J. Oncol..

[39] van der Bij G.J., Bögels M., Oosterling S.J., Kroon J., Schuckmann D.T., de Vries H.E., Meijer S., Beelen R.H., van Egmond M. (2008). Tumor infiltrating macrophages reduce development of peritoneal colorectal carcinoma metastases. Cancer Lett..

[40] Ueda H., Yamazaki C., Yamazaki M. (2003). Inhibitory effect of Perilla leaf extract and luteolin on mouse skin tumor promotion. Biol. Pharm. Bull..

[41] Plaschke K. Composition comprising one or more flavonoids, method of obtaining such composition and use thereof as UV-absorbing agent. US Pat..

[42] Gers-Barlag H., Kummerfeld D.E., Knüppel A., Müller A., Rümpel D.E., Stäb F., Dörschner A., Schönrock U. (2000). Wirkstoffkombination aus sulfonierten UV-Filtersubstanzen und Flavonderivaten und/oder Flavanonderivaten, insbesondere Flanonoiden, sowie kosmetische Zubereitungen, solche Wirkstoffkombinationen enthaltend. Pat. Appl. DE 199 23 712 A 1.

[43] Horváthová K., Novotný L., Vachálková A. (2003). The free radical scavenging activity of four flavonoids determined by the comet assay. Neoplasma.

[44] Horváthová K., Novotný L., Tóthová D., Vachálková A. (2004). Determination of free radical scavenging activity of quercetin, rutin, luteolin and apigenin in H2O2-treated human ML cells K562. Neoplasma.

[45] Horváthová K., Chalupa L., Sebova D., Tóthová D., Vachálková A. (2005). Protective effect of quercetin and luteolin in human melanoma HMB-2 cells. Mut. Res..

[46] Noroozi M., Angerson W.J., Lean M.E. (1998). Effects of flavonoids and vitamin C on oxidative DNA damage to human lymphocytes. Am. J. Nutr..

[47] Steffan B. (2005). Inhaltsstoffe aus Pflanzen der indonesischen Volksmedizin (Jamu): Isolierung, Identifizierung, und Charakterisierung der antioxidativen Eigenschaften. Doctoral thesis.

[48] Shimoi K., Masuda S., Shen B., Furugori M., Kinae N. (1994). Radioprotective effect of antioxidative flavonoids in gamma-ray irradiated mice. Carcinogenesis.

[49] Sharma V., Mishra M., Ghosh S., Tewari R., Basu A., Seth P., Sen E. (2007). Modulation of interleukin-1beta mediated inflammatory response in human astrocytes by flavonoids: implications in neuroprotection. Brain Res. Bull..

[50] Samy R.P., Gopalakrishnakone P., Ignacimuthu S. (2006). Anti-tumor promoting potential of luteolin against 7,12-dimethylbenz(a)anthracene-induced mammary tumors in rats. Chem. Biol. Interact..

[51] Manju V., Nalini N. (2005). Chemopreventive potential of luteolin during colon carcinogenesis induced by 1,2-dimethylhydrazine. Ital. J. Biochem..

[52] Choi J.S., Park K.Y., Moon S.H., Rhee S.H., Young H.S. (1994). Antimutagenic effect of plant flavonoids in the Salmonella assay system. Arch. Pharm. Res..

[53] Taj S., Nagarajan B. (1996). Inhibition by quercetin and luteolin of chromosomal alterations induced by salted, deep-fried fish and mutton in rats. Mutat. Res..

[54] Li Y.C., Hung C.F., Yeh F.T., Lin J.P., Chung J.G. (2001). Luteolin-inhibited arylamine N-acetyltransferase activity and DNA-2-aminofluorene adduct in human and mouse leukemia cells. Food Chem. Toxicol..

[55] Lu K.H., Lin K.L., Yang C.C., Hsia T.C., Hsiao Y.M., Chou M.C., Ho H.C., Chung J.G. (2002). The effect of paclitaxel on gene expression and activity of arylamine N-acetyltransferase and DNA-2-aminofluorene adduct formation in human leukemia HL-60 cells. Food Chem. Toxicol..

[56] Chen J.C., Chung J.G., Lin K.M. (2000). Effects of luteolin on arylamine N-acetyltransferase activity in human liver tumour cells. Cytobios.

[57] Su C.C., Chen G.W., Yeh C.C., Yang M.D., Hung C.F., Chung J.G. (2003). Luteolin induces N-acetylation and DNA adduct of 2-aminofluorene accompanying N-acetyltransferase activity and gene expression in human bladder cancer T24 cell line. Anticancer Res..

[58] Lee W.J., Wu L.F., Chen W.K., Wang C.J., Tseng T.H. (2006). Inhibitory effect of luteolin on hepatocyte growth factor/scatter factor-induced HepG2 cell invasion involving both MAPK/ERKs and PI3K-Akt pathways. Chem. Biol. Interact..

[59] Lansky E.P., Harrison G., Froom P., Jiang W.G. (2005). Pomegranate (Punica granatum) pure chemicals show possible synergistic inhibition of human PC-3 prostate cancer cell invasion across Matrigel. Invest. New Drugs.

[60] Yamashita N., Kawanishi S. (2000). Distinct mechanisms of DNA damage in apoptosis induced by quercetin and luteolin. Free Radic. Res..

[61] Cantero G., Campanella C., Mateos S., Cortés F. (2006). Topoisomerase II inhibition and high yield of endoreduplication induced by the flavonoids luteolin and quercetin. Mutagenesis.

[62] Sugimura T., Nagao M., Matsushima T. (1977). Mutagenicity of flavone derivatives. Proc. Jpn. Acad..

[63] van der Hoeven J.C.M., Bruggeman I.M., Debets F.M.H. (1984). Genotoxicity of quercetin in cultured mammalian cells. Mutat. Res..

[64] Chowdhury A.R., Sharma S., Mandal S., Goswami A., Mukhopadhyay S., Majumder H.K. (2002). Luteolin, an emerging anti-cancer flavonoid, poisons eukaryotic DNA topoisomerase. Biochem. J..

[65] Ruiz P.A., Haller D. (2006). Functional diversity of flavonoids in the inhibition of the proinflammatory NF-kappaB, IRF, and Akt signaling pathways in murine intestinal epithelial cells. J. Nutr..

[66] Agullo G., Gamet-Payrastre L., Manenti S., Viala C., Rémésy C., Chap H., Payrastre B. (1997). Relationship between flavonoid structure and inhibition of phosphatidylinositol 3-kinase: a comparison with tyrosine kinase and protein kinase C inhibition. Biochem. Pharmacol..

[67] Liu W., Liang N.C., Huang R.B. (2006). Effect of three flavones on enzyme activity of recombinant human phosphoinositide 3-kinase p110beta catalytic subunit [Article in Chinese]. Zhong Yao Cai.

[68] Shi R., Huang Q., Zhu X., Ong Y.B., Zhao B., Lu J., Ong C.N., Shen H.M. (2007). Luteolin sensitizes the anticancer effect of cisplatin via c-Jun NH2-terminal kinase-mediated p53 phosphorylation and stabilization. Mol. Cancer Ther..

[69] Han H.Y., Shan S., Zhang X., Wang N.L., Lu X.P., Yao X.S. (2007). Down-regulation of prostate specific antigen in LNCaP cells by flavonoids from the pollen of Brassica napus L.. Phytomedicine.

[70] Cheng A.C., Huang T.C., Lai C.S., Pan M.H. (2005). Induction of apoptosis by luteolin through cleavage of Bcl-2 family in human leukemia HL-60 cells. Eur. J. Pharmacol..

[71] Lee H.J., Wang C.J., Kuo H.C., Chou F.P., Jean L.F., Tseng T.H. (2005). Induction apoptosis of luteolin in human hepatoma HepG2 cells involving mitochondria translocation of Bax/Bak and activation of JNK. Toxicol. Appl. Pharmacol..

[72] Horinaka M., Yoshida T., Shiraishi T., Nakata S., Wakada M., Nakanishi R., Nishino H., Matsui H., Sakai T. (2005). Luteolin induces apoptosis via death receptor 5 upregulation in human malignant tumor cells. Oncogene.

[73] Michels G., Wätjen W., Niering P., Steffan B., Thi Q.H., Chovolou Y., Kampkötter A., Bast A., Proksch P., Kahl R. (2005). Pro-apoptotic effects of the flavonoid luteolin in rat H4IIE cells. Toxicology.

[74] Shi R.X., Ong C.N., Shen H.M. (2005). Protein kinase C inhibition and x-linked inhibitor of apoptosis protein degradation contribute to the sensitization effect of luteolin on tumor necrosis factor-related apoptosis-inducing ligand-induced apoptosis in cancer cells. Cancer Res..

[75] Lupu R., Menendez J.A. (2006). Pharmacological inhibitors of Fatty Acid Synthase (FASN)--catalyzed endogenous fatty acid biogenesis: a new family of anti-cancer agents?. Curr. Pharm. Biotechnol..

[76] van Zanden J.J., Geraets L., Wortelboer H.M., van Bladeren P.J., Rietjens I.M.C.M., Cnubben N.H.P. (2004). Structural requirements for the flavonoid-mediated modulation of glutathione S-transferase P1-1 and GS-X pump activity in MCF7 breast cancer cells. Biochem. Pharmacol..

[77] Graefe E.U., Derendorf H., Veit M. (1999). Pharmacokinetics and bioavailability of the flavonols quercetin in humans. Int. J. Clin. Pharmacol. Ther..

[78] (2007). USDA Databank for the flavonoid content of selected foods, 2^nd^ release. http://www.ars.usda.gov/nutrientdata.

[79] Bosetti C., Spertini L., Parpinel M., Gnagnarella P., Lagiou P., Negri E., Franceschi S., Montella M., Peterson J., Dwyer J., Giacosa A., La Vecchia C. (2005). Flavonoids and breast cancer risk in Italy. Cancer Epidemiol. Biomarkers Prev..

[80] Peterson J., Lagiou P., Samoli E., Lagiou A., Katsouyanni K., La Vecchia C., Dwyer J., Trichopoulos D. (2003). Flavonoid intake and breast cancer risk: a case--control study in Greece. Br. J. Cancer.

[81] Fink B.N., Gaudet M.M., Britton J.A., Abrahamson P.E., Teitelbaum S.L., Jacobson J., Bell P., Thomas J.A, Kabat G.C., Neugut A.I., Gammon M.D. (2006). Fruits, vegetables, and micronutrient intake in relation to breast cancer survival. Breast Cancer Res. Treat..

[82] Fink B.N., Steck S.E., Wolff M.S., Britton J.A., Kabat G.C., Gaudet M.M., Abrahamson P.E., Bell P., Schroeder J.C., Teitelbaum S.L., Neugut A.I., Gammon M.D. (2007). Dietary Flavonoid Intake and Breast Cancer Survival among Women on Long Island. Cancer Epidemiol. Biomarkers Prev..

[83] Rossi M., Negri E., Talamini R., Bosetti C., Parpinel M., Gnagnarella P., Franceschi S., Dal Maso L., Montella M., Giacosa A., La Vecchia C. (2006). Flavonoids and Colorectal Cancer in Italy. Cancer Epidemiol. Biomarkers Prev..

[84] Bosetti C., Rossi M., McLaughlin JK, Negri E., Talamini R., Lagiou P., Montella M., Ramazzotti V., Franceschi S., LaVecchia C. (2007). Flavonoids and the risk of renal cell carcinoma. Cancer Epidemiol. Biomarkers Prev..

[85] Garcia-Closas R., Agudo A., Gonzalez C.A., Riboli E. (1998). Intake of specific carotenoids and flavonoids and the risk of lung cancer in women in Barcelona, Spain. Nutr. Cancer.

[86] Neuhouser M.L. (2004). Dietary flavonoids and cancer risk: evidence from human population studies. Nutr. Cancer..

[87] Gates M.A., Tworoger S.S., Hecht J.L., De Vivo I., Rosner B., Hankinson S.E. (2007). A prospective study of dietary flavonoid intake and incidence of epithelial ovarian cancer. Int. J. Cancer.

